# Dual Characteristics of Apoptosis and Survival in Cerebellar Purkinje Cells Four Days After Transient Global Ischemia

**DOI:** 10.3390/cells15070572

**Published:** 2026-03-24

**Authors:** Zhen He, Alena Savenka, Ada G. Cino Ozuna, Kelly J. Davis, Angel Paredes

**Affiliations:** 1 Division of Neurotoxicology, HFT-132, National Center for Toxicological Research, Food and Drug Administration, 3900 NCTR Road, Jefferson, AR 72079, USA; 2 Nanotechnology Core Facility, National Center for Toxicological Research, Food and Drug Administration, Jefferson, AR 72079, USA; 3 Toxicologic Pathology Associates, National Center for Toxicological Research, Food and Drug Administration, Jefferson, AR 72079, USA

**Keywords:** non-canonical apoptosis, cerebellar Purkinje cells, global ischemia, mitochondria fragmentation and loss, nuclear apoptotic body, nuclear membrane blebbing, TUNEL-positive

## Abstract

Whether apoptotic cell death occurs in cerebellar Purkinje cells following transient global ischemia remains unclear. Histologic, immunofluorescent and ultramicroscopic methods were used to assess ischemic outcomes in rats. A pilot study using fluorescence labeling [TUNEL-caspase 3-activated peptide (C3AP)-DAPI] revealed key apoptotic characteristics including nuclear TUNEL-positive, nuclear membrane blebs (NMBs), and cytoplasmic C3AP-positive structures, in addition to transport of TUNEL-positive round structures into cytoplasm and projections in ischemic Purkinje cells but not in controls 4 days after ischemia. A formal follow-up study confirmed that 8% of Purkinje cells 4 days following ischemia exhibited TUNEL-positivity and/or NMBs, while Purkinje cells in controls did not. TUNEL-positive Purkinje cells displayed reduced intensity of Calbindin D28K-ifl/MitoTracker (living cell markers) labeling as compared to controls (*p* < 0.01). Ultramicroscopic evidence of apoptosis included mitochondrial fragmentation and loss in addition to NMBs and cytosolic deposit of nuclear autophagosomes. Interestingly, 71% of the Purkinje cells exhibited autophagy activity after ischemia. Ultramicroscopic characteristics of survival in ischemic Purkinje cells included a centrally located nucleus, no significant chromatin condensation, stable nuclear and intact cytoplasmic membranes, and normal peripheral spacing of the Purkinje cells. In conclusion, four days after transient global ischemia, cerebellar Purkinje cells exhibited both apoptotic and survival characteristics. Further research warrants investigation of the underlying mechanisms.

## 1. Introduction

Cerebellar Purkinje cells may die of ischemic attack. Ischemic damage has been identified with Fluoro-Jade B labeling of Purkinje cells 1- and 3-days post cardiac arrest and cardiopulmonary resuscitation (CA/CPR) [1]. Additionally, terminal deoxynucleotidyl transferase dUTP nick-end labeling (TUNEL) assessment was successful in identifying dying Purkinje cells in cerebellar Purkinje cell in vitro cultures [2]. However, the TUNEL approach, to the best of our knowledge, has not been successful in identifying the occurrence of apoptosis in cerebellar Purkinje cells in vivo following cerebellar ischemia. For example, TUNEL-positive cells have been identified in the cerebellar Purkinje cell layer in dogs following transient global ischemia; however, using an ultramicroscopic approach, the authors concluded that apoptotic cerebellar granular neurons were responsible for TUNEL signaling [1,3], rather than Purkinje cells. Quillinan et al. observed TUNEL-positive cells in the hippocampal CA1 region but failed to identify concurrent Purkinje cell damage on day 1, 3 and 7 post transient global ischemia in the CA/CPR mouse model [1]. Autopsy studies of individuals who survived from less than 1 h to 186 days (mean = 11 days) after a single cardiac arrest displayed a delayed, likely non-apoptotic, Purkinje cell death [4]. Using a specific in situ nick-end labeling method for DNA degradation, evidence of DNA fragmentation was found in the granular neurons but not in Purkinje cells of the cerebellum of patients that experienced transient global ischemia 4 or 10 days previously [5].

The goal of our study was to re-examine whether cerebellar Purkinje cells were subjected to apoptosis following transient global ischemia. A triple/quadruple fluorescent labeling method was employed to define apoptotic and living signs of the cerebellar Purkinje cells. First, a pilot study was launched using archived cerebral and cerebellar samples following transient global ischemia or sham-surgery [6,7,8,9,10,11]. After apoptotic characteristics in the pilot study were identified morphologically, immunohistochemically, and by TUNEL positivity, a formal follow-up study was launched using morphologic, immunohistochemical, TUNEL positivity and ultramicroscopic analysis.

## 2. Materials and Methods

Histological screening standards and process of immunofluorescent approach in the pilot study. Brain tissue blocks from adult and aged male Fischer 344 rats and adult female Sprague Dawley were collected from the previous studies [6,7,8,9,10,11]. The tissue sections included cerebellar tissue from sham-surgery/intact control groups (*n* = 7) and experimentally ischemic cerebellar tissue (transient global ischemia, *n* = 19). Specifically, the control group tissue sections were obtained from sham-surgery rats (total *n* = 5) involving adult male Fischer 344 rats (4-month-old): *n* = 2; an aged male (24-month-old) Fischer 344 rat: (*n* = 1); adult female Sprague Dawley rats: (*n* = 2); and intact aged (24-month-old) Fischer 344 rats (*n* = 2). Experimental group (ischemic) tissue sections were obtained from adult female Sprague Dawley rats (total *n* = 10) that underwent 20 min of transient global ischemia and survived for 2 days (*n* = 2) and 4 days (*n* = 4) post-ischemia or underwent 40 min of transient global ischemia and survived for 4 days (*n* = 2) or 8 days (*n* = 2) [6,7,8]. Notably, after 96 h of survival following ischemia, researchers observed ischemic damage and the effects of estrogen on cells expressing caspase-3 active peptide (the key executioner enzyme that drives apoptosis [12]) in the CA1 region of the hippocampus of adult female rats [6]. The remaining experimental samples (ischemic cerebellar tissue sections, *n* = 9 animals) were obtained from adult (4-month-old) or aged (24-month-old) male Fischer 344 rats that underwent 10 min of global ischemia, including two adult male rats and three aged male rats with a post-ischemic survival time of 3 days and two adult male rats and two aged male rats (a total of 4 animals, 1 animal/time point and age) with post-ischemic survival times of 8 and 14 days, respectively [9,10,11]. Screening criteria for ischemic cerebellar damage in the Purkinje cell layer were Purkinje cell loss and increased spacing between adjacent Purkinje cells; in the granular cell layer there was significantly increased pericellular space/cell-less space, perinuclear or whole-cell eosinophilic staining, interstitial eosinophilic staining, and/or decreased interstitial density. Furthermore, in the same animals, significant neuronal loss in the hippocampal CA1 region was also confirmed. The pilot study employed a triple fluorescence assay [11] with TUNEL-caspase 3 active peptide (C3AP)-4′,6-diamidinyl-2-phenylindole (DAPI) to analyze the cerebellar damage following transient global ischemia. “In Situ Cell Death Detection Kit, Fluorescein (green, fluorescent signal)” (Sigma-Aldrich, St. Louis, MO, USA) was employed as previously described [11]. Red fluorescent signal was used to tag C3AP (primary rabbit anti-C3AP antibody, R&D Systems, Minneapolis, MN, USA). Fluorescent goat anti-rabbit antibody (Molecular Probes/Invitrogen, Carlsbad, CA, USA) served as the secondary antibody for finalizing C3AP. A blue, fluorescent nucleic acid staining agent, 4′,6-diamidino-2-phenylindole (DAPI), was used to delineate the cellular nucleus. Each immunofluorescence labeling experiment involved placing eight or fewer tissue sections (one section/glass slide). To ensure reagent efficacy, the first few rounds of experiments included negative controls (cerebellar sections from sham-surgery and/or intact groups) and positive controls (ischemic hippocampal sections containing the CA1 region, previously validated in the triple-fluorescent labeling of TUNEL-C3AP-DAPI [11]), in addition to the ischemic cerebellar tissue sections to be tested. Purkinje cells were identified by their larger size (approximately 20–40 μm in diameter), spacious cytoplasm, and characteristic single-layer arrangement. The images obtained were used for institutional research protocol applications to facilitate subsequent studies. No further statistical analysis was performed other than recording the apoptotic characteristics (TUNEL- and C3AP-positive or negative) to filter out time points showing negative results.

Procedures and processes for the formal follow-up study. All animal procedures were approved by the National Center for Toxicological Research (NCTR) Institutional Animal Care and Use Committee and conducted in full accordance with the PHS Policy on Humane Care and Use of Laboratory Animals. Adult female Sprague Dawley rats (*n* = 6) weighing 245~290 g were purchased from Charles River Laboratories and acclimated for 1 week before surgical induction of transient global ischemia. Rats were pair-housed conventionally during acclimation and individually housed after the first day of surgery under a daily 12 h light cycle. All animals before and after surgery were allowed free access to food and water. In this formal follow-on study, two rats experienced sham surgeries, including permanent bilateral vertebral artery occlusion (BVAO) on Day 1 and sham surgery of bilateral carotid artery occlusion (BCAO) on Day 2. In the experimental group, four rats endured 20 min of transient global ischemia induced by 4VO, namely permanent BVAO on Day 1 and temporary BCAO on Day 2. Both groups of animals were sacrificed 4 days after the Day 2 surgery. To collect tissue, animals were exposed to a concentration of isoflurane (up to 4%) until a surgical plane of anesthesia was achieved. Animals were then euthanized by exsanguination via cutting of the right atrium of the heart after the chest cavity was opened. This was followed by an intra-arterial perfusion of 100 mL of saline followed by 100 mL of 4% paraformaldehyde-PBS buffer via the ascending aorta while the descending aorta below the diaphragm was occluded using vascular forceps. The whole procedure took 5~10 min.

Histological Methods. A forebrain block (3 mm-thick tissue block located 5.5 mm from the frontal pole) and a hindbrain block (cerebellum together with brain stem underneath cut half coronally) were taken from each brain and fixed in a 4% paraformaldehyde-PBS buffer. Both brain blocks were paraffin-embedded and coronally sectioned into 5 μm slices. Adjacent tissue sections (within 5- to 50-μm from each other) were microscopically evaluated by the hematoxylin and eosin (H&E) method and a triple/quadruple immunofluorescent labeling method.

Immunofluorescent Methods. Tissue sections were processed for fluorescent labeling and image acquirement. The images captured within the same-time fluorescent experiment were utilized for data analysis. First, all tissue sections were deparaffinized and rehydrated. Thereafter, a quadruple fluorescent labeling method [9] was utilized to visualize DNA degradation, nucleus, and cellular organelle of interest. Briefly, a direct TUNEL labeling assay was performed using “In Situ Cell Death Detection Kit, Fluorescein (green, fluorescent signal)” (Sigma-Aldrich, St. Louis, MO, USA) as previously described [11]. Red fluorescent signal was employed to tag mitochondria (MitoTracker™ Red CMXRos, M7512, Sigma-Aldrich, St. Louis, MO, USA) and/or the primary rabbit anti-CB28 antibody (Sigma-Aldrich, St. Louis, MO, USA) in the separated experiments. The fluorescent goat anti-rabbit antibody (Molecular Probes/Invitrogen, Carlsbad, CA, USA) served as the secondary antibody for finalizing CB28 labeling. Finally, a blue fluorescent nucleic acid-staining agent, DAPI, was used to delineate the cellular nucleus. For 100× amplification, images on TUNEL (green, fluorescent signal), C3AP immunoreactivity/MitoTracker™ Red/CB28 immunoreactivity (red, fluorescent signal), or DAPI staining (blue, fluorescent signal) were acquired using appropriate filters in a Zeiss AxioImager M2 microscope system with Software Zen 2. Stack images were acquired at 0.5-micron interval along the “Z” axis/longitudinal axis of the brain to generate 3-dimensional views/videos of the subcellular structures of interest.

For 10× and 40×/60× amplification, images with TUNEL, MitoTracker red-CB28 immunoreactivity, or DAPI staining were acquired using appropriate filters in Olympus BX40 microscope with Olympus CellSens software (version 4.4.1, Evident Scientific, State College, PA, USA). Image J (version 1.54p, National Institutes of Health (NIH), Bethesda, MD, USA) was used to measure the intensities of the MitoTracker-red-CB28 immunoreactivity in single-color images.

## 3. Analysis

The determined values, minus the background, divided by the determined cellular area were used for statistical analysis. GraphPad Prism 9 (GraphPad Software, LLC, Boston, MA, USA) Ordinary One-way ANOVA including Brown–Forsythe test and Bartlett’s test was applied to compare between three groups of cells: sham-surgery Purkinje cells (*n* = 20 or 10 Purkinje cells/animal), ischemic Purkinje cells without TUNEL-positive labeling (*n* = 20 or 5 Purkinje cells/animal), and ischemic Purkinje cell exhibiting TUNEL-positive tags (*n* = 20/24 total TUNEL-positive cells pooled from 4 animals). *p* < 0.05 was considered as significant. Purkinje cells exhibiting a red fluorescent signal (Mitotracker marker/CB28-ifl) were counted using 10× images and verified using 40×, 60×, and/or 100× images. The 10× microscopic view field was measured with scale and Image J software to count cell number per area. TUNEL-positive cells that also showed nuclear membrane blebbing were counted as damaged cells, in which the apoptotic process was considered “initiated”.

Transmission electron microscopy (TEM) approach. The cerebellar tissue blocks (sham surgery control group and experimental ischemic group) previously stored in 4% paraformaldehyde PBS were sliced into multiple small blocks of a size 1 × 1 × 1 mm^3^ and reprocessed for electron microscopy. These blocks were washed in 0.15 M cacodylate buffer, pH 7.4, and then refixed in 0.15 M cacodylate, 2.5% glutaraldehyde, 2 mM calcium chloride. The staining and embedding protocol followed the serial block face SEM (SBF-SEM) procedure [13].

After polymerizing in Durcupan resin, the blocks were thin-sectioned using a Leica UC-6 ultramicrotome and imaged by TEM in a JEOL JEM-2100 electron microscope (Peabody, MA, USA) operating at 200 keV.

Serial block face-scanning electron microscopy (SBF-SEM). Five-micron-thick, cerebellar paraffin-embedded tissue sections on glass slides were used for the SBF-SEM analysis. The paraffin was removed using the procedure outlined by Cell Signaling Technology (IHC-P Protocol—for SignalStain Boost Detection Reagent|Cell Signaling Technology), and once deparaffinized, the sections were stained and embedded following the SBFSEM procedure [13]. The intended approach was to further explore the ultramicroscopic morphology in 3D of the Purkinje cells that were labeled TUNEL-positive and/or exhibited nuclear membrane blebbing. Because the Purkinje cells ranged ~20 micron or larger in size, the tissue sections either that displayed the TUNEL-immunofluorescent labeling or that sat closely adjacent to one containing the TUNEL-positive Purkinje cells were selected.

As before, the procedure included sample fixation, wash, incubation, re-staining and refixation, and dehydration before the Durcupan resin-embedding procedure. The embedded samples were sectioned 50 nm thick by a computer-controlled Gatan 3View2 serial block face microtome (Gatan, Inc., Pleasanton, CA, USA) on a Zeiss Merlin scanning electron microscope (Carl Zeiss, Oberkochen, Germany). Images were recorded between each section to generate 3D image stacks of the sample. Pixel sizes were determined by the settings set for the Gatan 3View2. Image stacks were then imported onto a Dell workstation and processed with Dragonfly image processing software Dragonfly 3D World (Comet Technologies, Shelton, CT, USA) to extract relevant structural information using computer-learning routine within Dragonfly.

## 4. Results

### 4.1. Pilot Study with a Triple Fluorescent Approach Exhibiting Apoptotic Characteristics of Purkinje Cells Following Transient Global Ischemia

**Figure 1:**
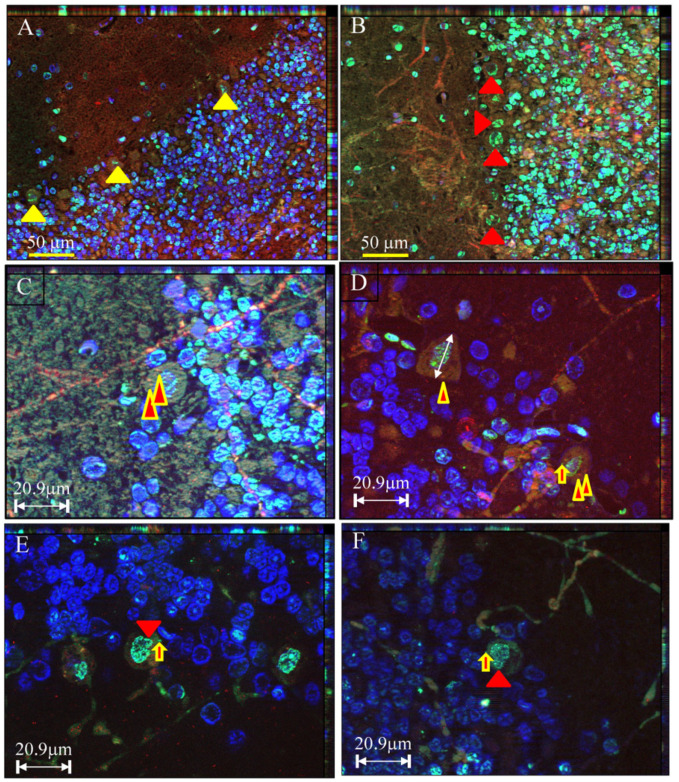
Triple fluorescent labeling of TUNEL (green, fluorescent signal), caspase-3 active peptide-immunoreactivity (C3AP-ir, red fluorescent signal), and nucleic labeling of DAPI displays apoptotic signs of the Purkinje cells following transient global ischemia. Purkinje cells can be identified by their larger size (approximately 20–40 μm in diameter), spacious cytoplasm, and characteristic single-layer arrangement ((**A**,**B**), arrowheads indicated). Purkinje cells subjected to apoptotic process may exhibit entire nucleic TUNEL-labeling with balloon-like nucleus (**A**) or partial nucleic TUNEL-labeling (**C**). “Apoptotic trilogy” is coined here to indicate simultaneous nucleic blebbing and TUNEL-positivity and cytosolic C3AP-ir ((**C**,**D**), two-color arrowhead indicated). TUNEL-positive bubble-like structures might deposit into cytosolic compartments ((**E**,**F**), two-color arrow indicated), called nucleic apoptotic bodies (NABs) due to their nature, less likely to include cellular plasma structures. Formation of NABs may indicate damage confinement and separation of the toxic, damaged molecules from cellular nucleus.

Characteristic apoptotic manifestations, i.e., apoptotic trilogy including co-localization of nuclear TUNEL positivity and DAPI nuclear markers, nuclear membrane blebbing, and cytoplasmic C3AP expression, were all observed in Purkinje cells of animals surviving for 4 days after transient ischemia (*n* = 6) (Figure 1). Among other post-ischemic survival periods, i.e., post-ischemia 2, 3, 8 and 14 days, only a single apoptotic Purkinje cell (nuclear TUNEL-positive and nuclear membrane blebbing) was identified 3 days following the onset of ischemia in an aged (24-month-old) Fischer 344 male rat (*n* = 1). No such manifestations were found in the sham surgery or intact animals. Surprisingly, TUNEL-positive round structures were frequently observed in the cytoplasm and the projections (dendrite and axon) in the ischemic group (Figure 1).

Apoptotic CA1 neuronal death was confirmed according to the following criteria: TUNEL-positive labeling with or without C3AP-positive labeling in addition to condensed nuclear labeling (TUNEL-DAPI) and shrunken nucleus and cytosol space. Sixty-two Purkinje cells were found to exhibit nuclear TUNEL-positive labeling, nuclear membrane blebbing, and NABs deposited in cytosol, axon, and dendrite with or without C3AP immunoreactivity. None of the 62 TUNEL-positive Purkinje cells showed the critical morphologic signs of apoptosis, i.e., condensed nuclear labeling (TUNEL/DAPI) and shrunken nucleus and cytosol space (Figure 1).

### 4.2. Formal Follow-On Study, Through Histological Assessment, Revealed Cerebellar Ischemic Injury

**Figure 2:**
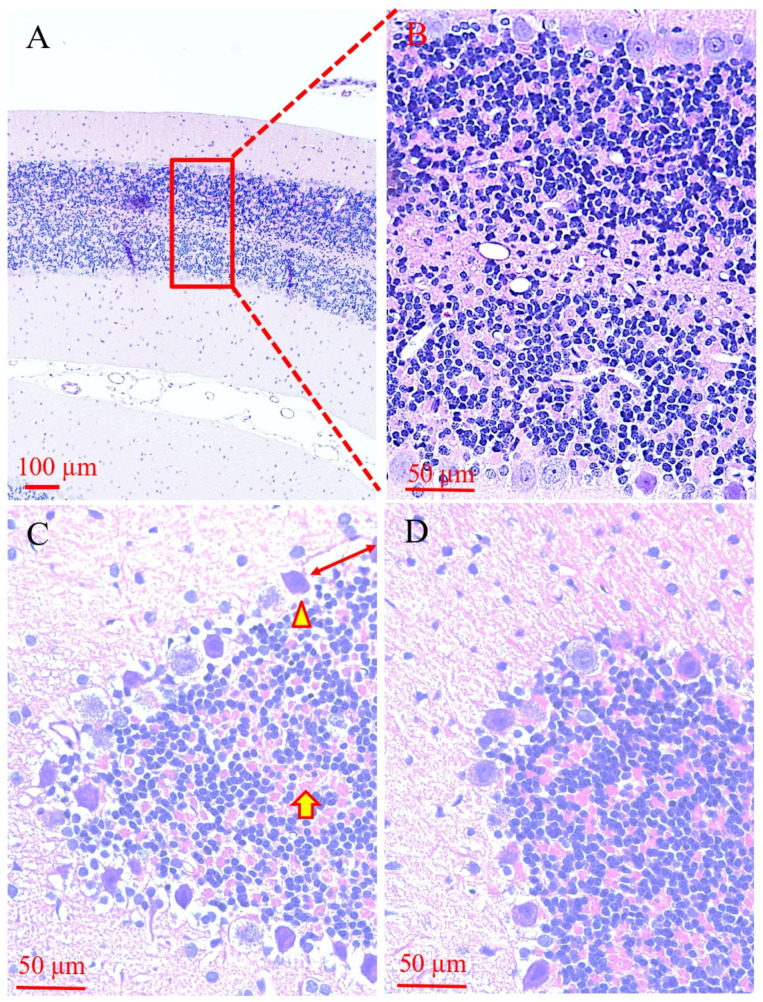
Histological (H&E staining) evidence of cerebellar damage following transient global ischemia in rats. Representative morphology of sham surgery control group is presented at low (10×, (**A**)) and high (40×, (**B**)) magnification. Purkinje cells can be identified by their larger size (approximately 20–40 μm in diameter), spacious cytoplasm, and characteristic single-layer arrangement (Purkinje cells arranged in lines at the top and bottom in (**B**)). Ischemic cerebellar (**C**,**D**) damage is verified by (1) reduced numbers of Purkinje cells/increase in the interval distance between two adjacent Purkinje cells (indicated by two direction arrows); (2) significantly increased peripheral cellular stainless space (arrowhead-indicated); (3) perinuclear or whole-cellular eosinophilia; (4) interstitial eosinophilia (arrow-indicated), and (5) reduced interstitial density.

As compared with the sham surgery control group (*n* = 2), rats subjected to 20 min of transient global ischemia (*n* = 4) displayed an increase in the interval distance between two adjacent Purkinje cells, significantly increased peripheral cellular empty/space, perinuclear or whole-cellular eosinophilia, interstitial eosinophilia (arrow-indicated), and/or reduced interstitial density (Figure 2).

### 4.3. Formal Follow-On Study with Immunofluorescent Assessment Highlighted Coexistence of Apoptotic Signals and Living Signs

**Figure 3:**
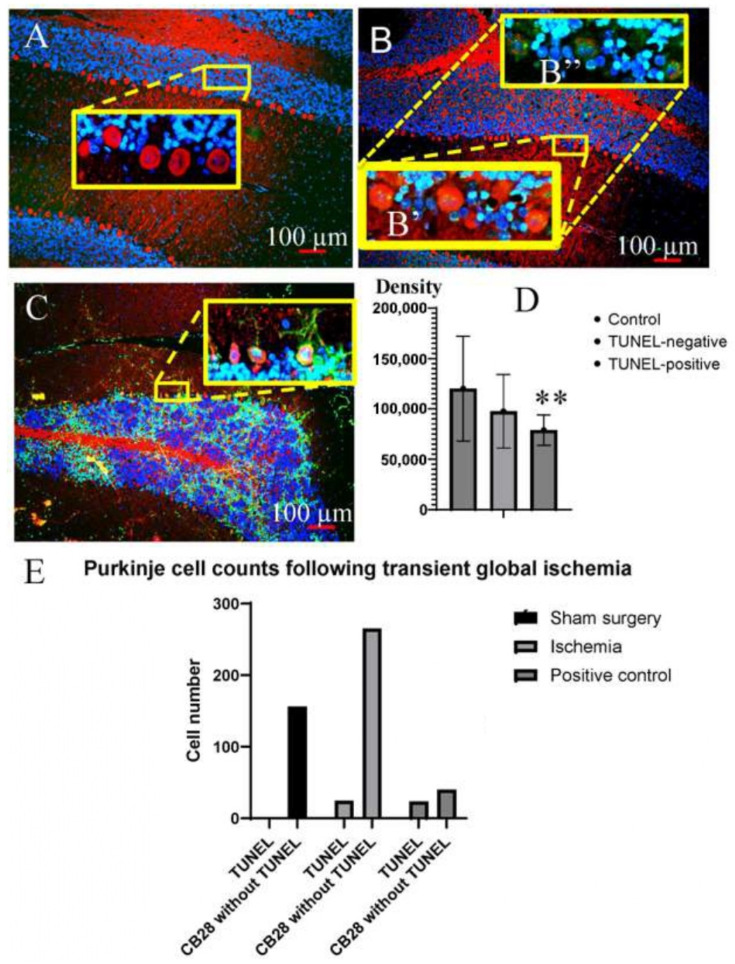
A triple fluorescent study addressing cerebellar Purkinje cell apoptotic (TUNEL, green) and/or living (MitoTracker-labeling) and/or CB28 (immunofluorescent labeling, red) parameters. Purkinje cells showing red fluorescent signal (Mitotracker label/CB28-ifl) were counted using 10× images in the sham surgery control group ((**A**), *n* = 2), ischemia group ((**B**), *n* = 4), and the retained sample was used as tissue-positive control ((**C**), *n* = 1). The TUNEL-positive Purkinje cells labeled with a red fluorescent signal were verified by 40× imaging, and the yellow patches indicate the green (TUNEL) signals overlapped with red signals (inserts (**B′**,**B″**)). By removing the red signals, the green signals/TUNEL-positive tags were confirmed to be colocalized with blue fluorescence/DAPI which outlined the nucleus (**B″**). Panel (**D**) exhibits comparison of fluorescent signal (red) intensities of mitochondria (MitoTracker-labeling) and/or CB28 (immunofluorescent labeling) between Purkinje cells in sham surgery control group (*n* = 20, left column), ischemic group without TUNEL-positive signal (*n* = 20, middle column) and ischemic group with TUNEL-positive signal (*n* = 20, right column). ** *p* < 0.01. Panel (**E**) shows the statistical results of the frequency of TUNEL-positive Purkinje cells in the sham surgery control group and the ischemia group.

As demonstrated in Figure 3A, Purkinje cells in the sham surgery control group exhibited extensive red fluorescent signal indicating expression of calbindin d28k (CB28)/mitochondria labeling without TUNEL labeling (green, fluorescent signal). A subset of the Purkinje cells subjected to transient global ischemia displayed nuclear TUNEL-positive signal and nuclear membrane blebbing (Figure 3B).

Measuring the fluorescent signal intensities of randomly selected sham surgery control group Purkinje cells (*n* = 20 or 10 Purkinje cells/animal), Purkinje cells experiencing transient global ischemia but displaying TUNEL negative signal (*n* = 20 or 5 Purkinje cells/animal), and Purkinje cells subjected to transient global ischemia and exhibiting TUNEL positivity (*n* = 20/24 total TUNEL-positive cells pooled from four animals) (Figure 3D, ANOVA summary: F = 5.988, [*p* < 0.01]) verified a reduction in intensity of CB28-ifl/MitoTracker labeling in the TUNEL-positive Purkinje cells as compared to the other two groups.

A total of 156 Purkinje cells were imaged and recorded from two sham surgery control group animals, and none of the cells showed TUNEL positivity (Figure 3A). On the other hand, among 289 Purkinje cells imaged and recorded from four transient ischemia animals, 8% (24) of Purkinje cells showed TUNEL positivity and nuclear membrane blebbing, with or without NAB deposition in their cytoplasm (Figure 3B). Using the cerebellum samples retained from previous studies [6,7,8,9,10,11] (*n* = 1) as a positive control, 23 of 63 Purkinje cells showed TUNEL positivity and nuclear membrane blebbing, with or without NAB deposition in their cytoplasm. Alternatively, Purkinje cell counts were 35 ± 7 mm^2^, 33 ± 7 mm^2^, and 21 ± 10 mm^2^, in the sham surgery control group, ischemic group, and positive control respectively (ANOVA summary: F [2,4] = 1.458 (*p* = 0.334). The double-fluorescence-labeling (CB28 + TUNEL) cell counts were 0 ± 0 mm^2^, 3 ± 2 mm^2^, and 8 ± 6 mm^2^ respectively in the sham surgery control group, ischemic group, and positive control (ANOVA summary: F [2,4] = 7.143 (*p* = 0.048). In addition to the existence of numerous TUNEL-positive granular cells indicating severe and extensive ischemic damage, some NABs were found in the secondary branches of dendrites and axons of the Purkinje cells (Figure 3C). Measurement of the frequency of TUNEL-positive cells among all Purkinje cells in the recorded images showed clear statistical significance (*p* < 0.001, Figure 3E). Nevertheless, none of the TUNEL-positive Purkinje cells exhibited key apoptotic features, namely nuclear condensation and reduction in nuclear or cytoplasmic space. On the other hand, in the positive control sample highlighting the CA1 region of the ischemic hippocampus, 27 CA1 neurons judged to be apoptotic showed TUNEL positivity with or without C3AP immunoreactivity and simultaneously displayed nuclear condensation and/or atrophy of the nucleus and cytoplasm.

**Figure 4:**
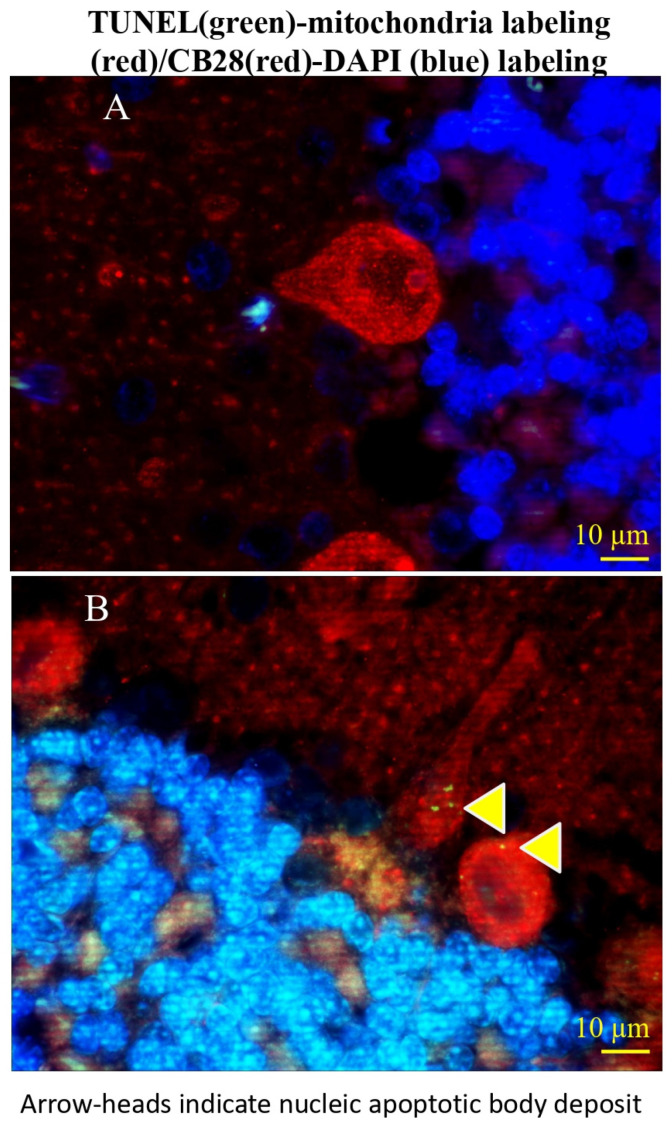
A triple fluorescent label displaying Purkinje cells and addressing both cell-living signs and cell-apoptotic signs in sham surgery control group (**A**) and ischemic group (**B**) in rats. Purkinje cells retain mitochondria and/or express calbindin D28K (CB28) (red, fluorescent signal), which indicates living signs, while simultaneously exhibiting as nucleic TUNEL (green, fluorescent signal)-positive, which signifies apoptotic signals. Arrowheads on low panel indicates transportation of nucleic apoptotic bodies (NABs) into Purkinje cell dendrite and cytosolic compartment.

TUNEL-positive round structures, mostly sized 1–2 microns in diameter, were found in cytosol or dendrites of some Purkinje cells (Figure 4). To acknowledge their TUNEL-positive characteristics and their nuclear origin, we coined these vesicle-like structures as nuclear apoptotic bodies (NABs).

### 4.4. Ultramicroscopic Findings Addressing Both Ischemic Damage and Evidence of Viability in Purkinje Cells

**Figure 5:**
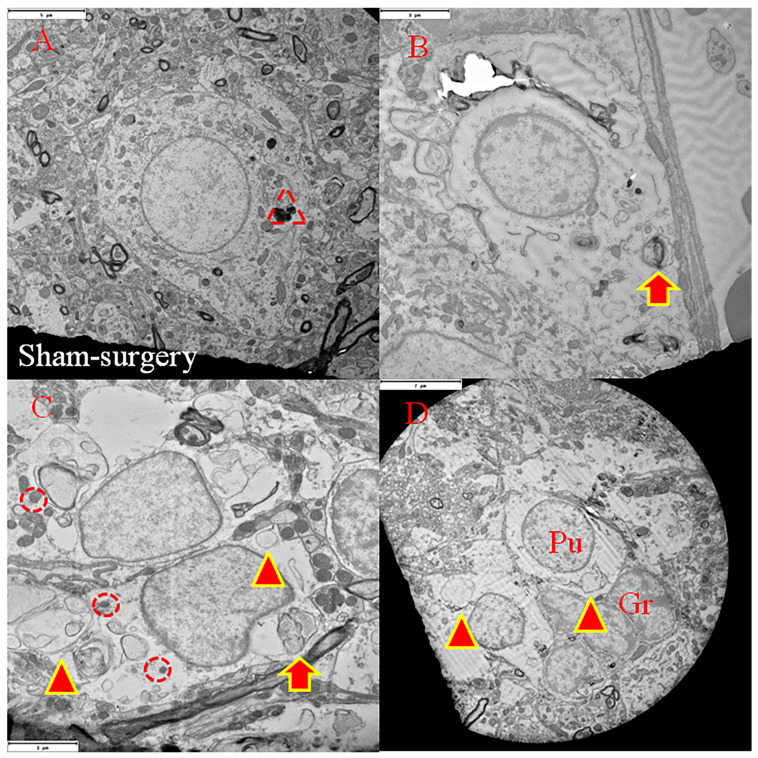
TEM micrographs demonstrating some injury with living characteristics in cerebellar Purkinje cells following transient global ischemia in rats. (**A**): Purkinje cell in sham surgery control group; (**B**–**D**): Purkinje cells with or without granular cells in the ischemic group. Features of injury of the Purkinje cells include accumulation of DNA along the nuclear membrane and degradation of cellular organelles like mitochondria and endoplasmic reticula. Characteristics of survival of the Purkinje cells following transient global ischemia involve centrally located nucleus, no significant chromatin condensation, intact and/or normal nucleic and cytoplasmic membranes, and normal peripheral space of the Purkinje cell(s). The dotted triangle indicates putative nuclear autophagosome (PNA) (Figure 4A). The dotted circle highlights mitochondrial fragmentation. Arrow specifies an unknown object, probably an autophagosome-/lysosome-like structure within which degraded Golgi apparatus/endoplasmic reticulum or mitochondria are present. Arrowhead indicates cytoplasmic vacuole-like structures. Pu: Purkinje cell. Gr: granular cell. (**A**): Scale bar = 5 μm, (**B**,**C**): Scale bar = 3 μm, (**D**): Scale bar = 7 μm.

In contrast to the sham surgery control group (Figure 5A), the representative electron microscopy of the cerebellar Purkinje cells subjected to ischemia clearly demonstrated cellular damage (Figure 5B–D). The cellular damage included accumulation of DNA along the nuclear membrane and degradation of cellular organelles like mitochondria and endoplasmic reticula. Interestingly, the fragmentation of mitochondria co-existed with cytoplasmic vacuole-like structures (Figure 5C). Despite these findings typically associated with cell damage, the nuclei of these Purkinje cells still occupied the central location of the cell body, and no significant chromatin condensation was present. In addition, the damaged Purkinje cells did not exhibit cellular shrinkage or enlargement. No apparent cellular deformation was detected. Finally, structures surrounding the cytoplasm, including the spaces directly outside of the cytoplasmic membranes attaching the synapses to the dendrites, appeared normal. Unexpectedly, a putative nuclear autophagosome (PNA), a form of autophagy, was found in a sham surgery control Purkinje cell (Figure 5A, red triangle-highlighted).

**Figure 6:**
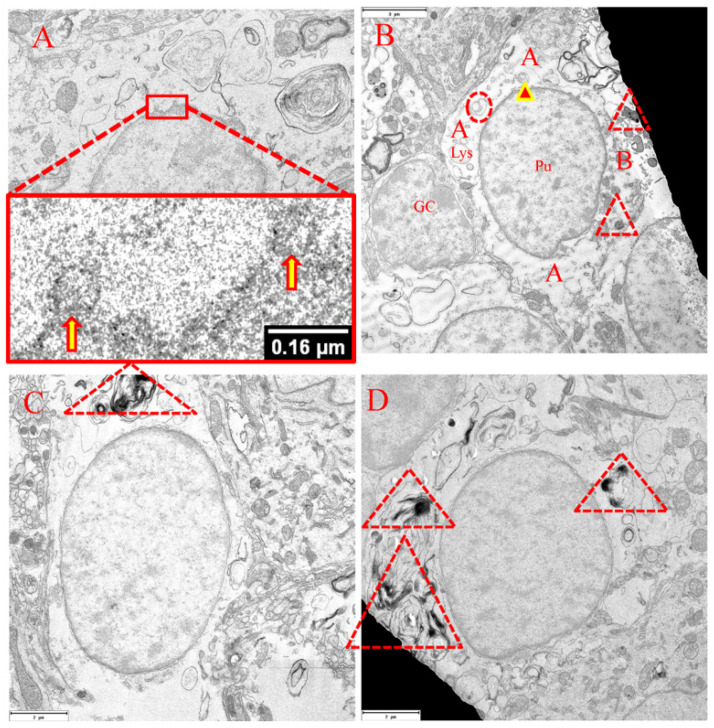
Ultramicroscopic evidence addressed the formation of nuclear apoptotic bodies and autophagic activities in rat cerebellar Purkinje cells following transient global ischemia. (**A**): nuclear membrane blebbing leads to the formation of nuclear apoptotic bodies (NABs), as indicated by arrow. (**B**): All necessary autophagic components could be observed within a Purkinje cell. Dashed circle indicates a phagophore; arrowhead highlights an autophagosome; Lys: autolysosome; dashed triangle contains putative nuclear autophagosome/NAB, which relatively demonstrates enhanced density. It appeared that Compartment A where autophagy occurred was different from Compartment B where cellular organelles were relatively less damaged. GC: granular cell, Pu: Purkinje cell. (**C**,**D**): various forms of putative nuclear autophagosomes/NABs are highlighted with dashed triangle. (**B**): Scale bar = 3 μm, (**C**,**D**): Scale bar = 2 μm.

In a separate portion of this study, all Purkinje cells were designated for imaging. In the ischemic group dedicated to TEM procedures, we counted a total of 45 Purkinje cells in three tissue blocks approximately 1 mm^3^ in size. Nuclear membrane blebbing was identified (Figure 6A). Notably, 32 Purkinje cells, or 71% of the total number of Purkinje cells, exhibited autophagic activity, involving phagophores, autophagosomes, autolysosomes, nuclear autophagosomes or PNA, and/or cytoplasmic vacuole(s) (Figure 6B). The remaining Purkinje cells consistently showed nuclear membrane invagination. Among 32 Purkinje cells, two showed not only autophagy activity but also nuclear membrane invagination. Various forms of PNA are visualized (Figure 6C,D).

**Figure 7:**
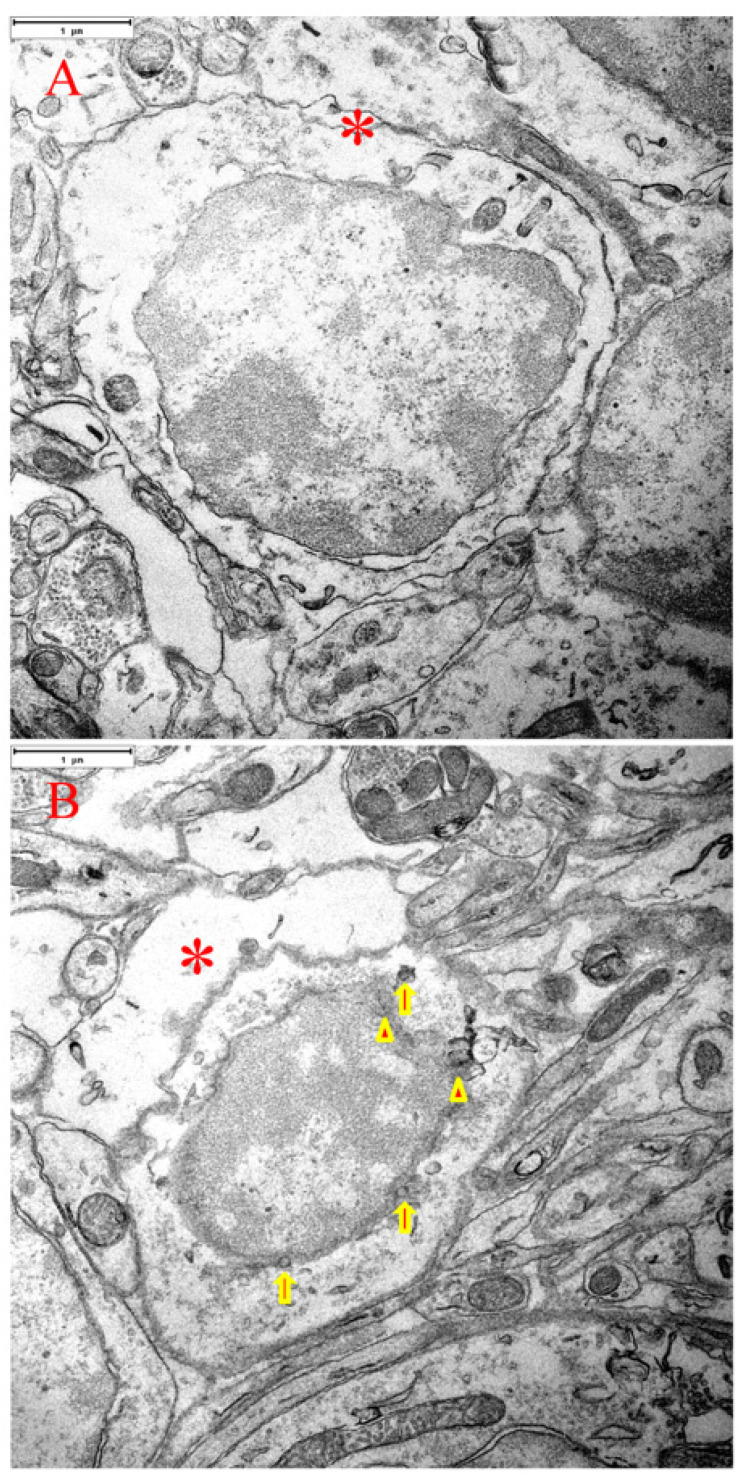
TEM micrographs demonstrating pro-apoptotic or apoptotic signs in cerebellar granular cells following transient global ischemia in rats. (**A**) A pro-apoptotic granular cell exhibits condensed chromatin, pyknotic nucleus, and relatively increased cytosolic space. (**B**) An apoptotic granular cell displays condensed chromatin, pyknotic nucleus, increased cytosolic space, nuclear membrane blebbing (arrowhead-specified) and nuclear apoptotic body formation (arrow-indicated), and increased cellular peripheral space (“*” indicates). (**A**,**B**): Scale bar = 1 μm.

Consistent with the study of Martin et al. [3], cerebellar granular cells undergoing apoptosis were identified by the following morphologic (EM) criteria: condensed chromatin and pyknotic nucleus, nuclear membrane blebbing and nuclear apoptotic body formation, relatively increased cytosolic space, and increased cellular peripheral space (Figure 7).

**Figure 8:**
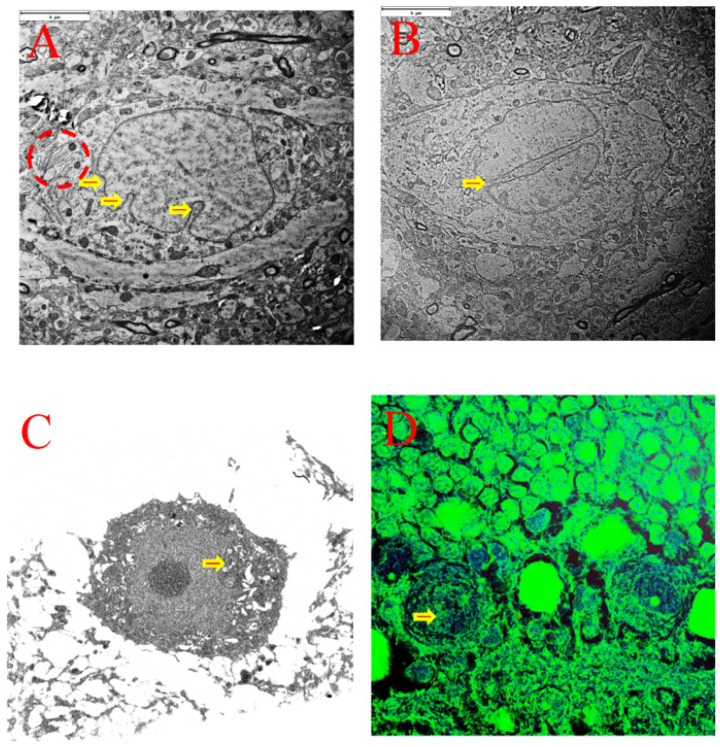
Electron microscopy images demonstrating nuclear membrane invaginations and lobulated nucleus in cerebellar Purkinje cells in rats of both sham surgery control group ((**A**,**B**), arrow indicated) and ischemic group ((**C**,**D**), arrow indicated). Various forms of nuclear membrane invaginations (NMIs) (**A**) and lobulated nucleus using TEM (**B**) and SBF-SEM ((**C**,**D**), arrow are seen). The dotted circle highlights annulate lamellae. The color can be arbitrarily selected by the software. (**A**,**B**): Scale bar = 4 μm.

Consistent with EM outcomes of various forms of nuclear membrane invaginations (Figure 8A) and lobulated nucleus (Figure 8B), SBF-SEM images 3-dimensionally verified the existence of nuclear membrane invagination (Figure 8C) and lobulated nucleus (Figure 8D).

## 5. Discussion

The present study extended the findings demonstrated by Martin et al. [3]. Supported by immunohistochemical and ultramicroscopic approaches, the novel findings in this study are that a subset of cerebellar Purkinje cells did exhibit time-limited, classic apoptotic changes following onset of ischemia, while these cells appeared not to be dead/dying of apoptosis. A detailed description of the apoptotic features is as follows: Up to four apoptotic hallmarks can be observed simultaneously in the same Purkinje cell (Figure 1D, including nuclear TUNEL positivity, nuclear membrane blebbing, formation of nuclear autophagosomes, and expression of active caspase 3 in the cytoplasm; or Figure 3C, including nuclear TUNEL positivity, nuclear membrane blebbing, formation of NABs/nuclear autophagosomes, and mitochondrial loss, the latter of which was confirmed in Figure 3D). Ultrastructural analysis confirmed the presence of nuclear membrane blebbing, NAB/nuclear autophagosome formation, and mitochondrial fragmentation/loss in ischemic-damaged Purkinje cells (Figure 5 and Figure 6). Therefore, unless these cells can recover from the ongoing apoptotic process, they will either die of apoptosis (presumably irreversible) or become senescent “zombie” cells [14].

In the follow-up official study of this report, we only investigated one time point of cerebellar Purkinje cell apoptosis (4 days after ischemia). However, studies in humans (less than 1 h to 186 days after ischemia) [4] and rodents (1, 3, and 7 days after transient global ischemia) [1] suggest that Purkinje cell death is a delayed process and may not be apoptotic cell death. Our pilot experiments, conducted on male and female rats of two strains, both young and old, using triple fluorescent labeling and observed from day 2 to day 14, allowed us to reproducibly investigate the apoptotic process of Purkinje cells on day 4 after transient global ischemia. It is highly likely that if Purkinje cell apoptotic death occurs, it will do so between day 4 and day 7 after transient global ischemia. Future studies may need to focus on this period.

### 5.1. Immunohistochemical Evidence Highlighting Both Apoptotic and Viability Manifestations of Purkinje Cells

We demonstrate here signs of apoptosis of the cerebellar Purkinje cells with the following evidence: TUNEL-positivity, nuclear morphologic features/nuclear membrane blebbing, and cytoplasmatic active caspase-3. Accordingly, we have little doubt that apoptosis was initiated in these cells. However, the expression of calbindin D28K (CB28) is a well-accepted sign of viable Purkinje cells [15]. In addition, mitochondria are essential for a living cell. Here we used a dual labeling method with a single color (red) of fluorescent signal labeling either mitochondria (MitoTracker-labeling) or CB28 (immunofluorescent labeling/ifl). Either one, if positive, suggests a cell in a living status, while TUNEL-positive signal (green fluorescence) was simultaneously applied to label ischemia-damaged Purkinje cells. As exhibited, the apoptotic signs and living manifestations co-exist in a subset (8%) of the cerebellar Purkinje cells subjected to 20 min of transient global ischemia, while severe ischemia may lead to the up-regulation of such cells (Figure 3C,E). In predictable apoptosis, cytoplasm membrane blebbing occurs before forming apoptotic bodies in which both nucleic and cytosolic components are involved. The nucleic membrane blebbing in TUNEL-positive Purkinje cells, but without cytoplasmic membrane blebbing, suggests several possibilities. One possibility is that the Purkinje cells may continue to display signs of life and continue to function until apoptosis is further progressed as compared to other cell types. If that is the case, an unknown process, one with neuro-preservative potential, may result in a time gap between the emergence of nuclear membrane blebbing and the occurrence of cytoplasmic membrane blebbing. Another possibility is that the launched apoptotic process may stop, and a rescue from the apoptotic processes may occur. It is hypothesized that the formation, deposition, and transport of NABs are a self-rescue process through which damaged, toxic portions of DNA are repaired or removed from the cell core (i.e., the nucleus).

### 5.2. Ultrastructural Evidence Suggests That Damaged Cerebellar Purkinje Cells May Have Self-Rescue Capabilities

Immunofluorescence Mitotracker/CB28 labeling showed that the intensity of Purkinje cells with TUNEL-labeling positivity was significantly reduced after ischemia (Figure 3D). Electron microscopy imaging results suggested that this decrease was due to the loss of cytoplasmic mitochondria (Figure 5B–D). Nonetheless, ultramicroscopic evidence did demonstrate cytosolic compartmentations (Figure 5B): one compartment showed autophagic activity and almost no mitochondria; the other compartment appeared “intact” and included many mitochondria, indicating that the mitochondrial compartmentation may have provided sufficient energy to the Purkinje cells.

Additional evidence supporting the existence of viability in these Purkinje cells included the transport of NABs from the nucleus to the cytoplasm and its cytoplasmic cell processes, such as Purkinje cell dendrites (Figure 3C and Figure 4B). In addition, no cellular shrinkage, a typical feature of apoptosis, was observed in the Purkinje cells. Hypothetically, one or more events occurred in these Purkinje cells and delayed or reversed the apoptotic cell death process. Previous research by others demonstrated that apoptotic cells may be rescued from cell death after an apoptotic stimulant is removed [16]. Early DNA repair could be involved in the reversal of the apoptotic process [17]. Additionally, Purkinje cells may be able to reverse the apoptotic process by nuclear membrane blebbing → NAB/nuclear autophagosome formation → NAB/nuclear autophagosome trafficking. The NAB/nuclear autophagosome trafficking may potentially be an active cellular mechanism that could play a role in separating the harmful degraded DNA from an affected Purkinje cell.

Of interest are the vacuole-like structures localized within a subset of the Purkinje cells following ischemia. These vacuole-like structures are morphologically comparable to the autophagosomes where the damaged organelles are conventionally digested by lysosomes. All necessary autophagy components have been verified, including phagophores, autophagosomes, autolysosomes, and nuclear autophagosomes (Figure 5A and Figure 6). Programmed cell death includes apoptosis (Type I), autophagy (Type II), and necrosis (Type III) [18]. Of note is the process of autophagy in which the membrane-bound structures, i.e., autophagosomes, remain within the cytosol. “Autophagy is defined as the lysosomal degradation of intracellular components” [19]. It is likely that when the post-ischemic Purkinje cell is alive but struggling to survive, an autophagy process is activated once the first NAB deposits into the cytosol. Briefly, the signaling pathway of the crosstalk between apoptosis and autophagy displays the following: phagophore formation → phagophore expansion → autophagosome → inhibition of apoptosis [19]. The formed autophagosomes may critically affect the host cell’s fate, to die or to survive.

Interestingly, we demonstrated nuclear membrane blebbing in association with formation of NABs on the nuclear membrane in a cerebellar granular cell following transient global ischemia via EM image analysis (Figure 7B). The formed NABs deposited into the cytosol simultaneously exhibited a compromised/broken nuclear membrane (Figure 7B). Shrinkage of its cytosol and nucleus strengthens the odds of a death process for this granular cell. Supposing that a neuroprotective mechanism is Purkinje cell-specific, EM imaging should exhibit a clear difference from that displayed in cerebellar granular cells.

Evidence from two sources indicates that cerebellar Purkinje cells exhibit both apoptotic and survival characteristics in response to transient global ischemia and this phenomenon is cell-type specific. First, inflammation delays apoptotic cell death of neutrophils [20,21,22], and conventionally mature neutrophils nuclei are lobulated [23]. Coincidentally, various forms of nuclear membrane invaginations and existence of the lobulated nucleus are readily definable in the cerebellar Purkinje cells both in the sham surgery control group and animals subjected to transient global ischemia. This was observed with both standard 2D transmission electron microscopy (TEM) as well as serial block face-scanning electron microscopy (SBFSEM) which reproduces the tissue in 3D (Figure 8), both of which sampled a limited cerebellar tissue volume and/or a limited Purkinje cellular volume. In addition, the present study demonstrates that Purkinje cells exhibiting multiple nuclear membrane invaginations (NMIs) may simultaneously display the annulate lamellae (AL) structure (Figure 7A). The AL pore complexes (ALPCs) contain almost all nuclear pore complex (NPC) proteins in addition to the similarity in morphology between ALPCs and NPC [24,25]. The ALPCs interact with the nuclear transportation complexes potentially via reverse regulation of the number of NPCs and modification of a nuclear Ras (Rat sarcoma virus)-like GTPase [25,26]. On the other hand, it is known that part of the NMIs constitutes nucleoplasmic reticulum (NR) [26]. Pleomorphic forms of NMIs may indicate differential functions and behavior of each of the NMIs [27]. Interestingly, NMIs are linked to lobulated nuclei when lamins with the CaaX motif [28] overexpress and the nuclear envelope is extended [29,30]. Of interest is that occurrence of cerebral ischemia is followed by a marked inflammatory reaction [31] manifested by expression of cytokines and other inflammatory mediators and inflammatory cell-infiltration as well. Perhaps the morphologic characteristics of the lobulated nucleus may afford a barrier between the lobulations of the nucleus and deter the transmission of the damaging/apoptotic signals derived from ischemia.

Hypothetically, another potential neuroprotective mechanism may exist. It is known that apoptosis does not occur in cells undergoing endoreplication [32], which may be applicable to cerebellar Purkinje cells. First, cellular body weight increase is concomitantly associated with enlargement of the cell body in a subset of neurons in which endoreduplication of the DNA and enhancement of certain gene expressions occur in adult mollusks [33]. Notably, cerebellar Purkinje cells are polyploid [34], attributable to the fact that a mature Purkinje neuron establishes one of the most extensive connections with up to ~200,000 granular neurons in the cerebellum [35] in addition to the direct contacts with the climbing fibers and links from the Golgi, stellate, and basket cells as well. As estimated, each Purkinje cell may collect more than 1 million signals from other neurons [36]. The microspectrophotometric analysis of single human Purkinje neurons indicates the tetraploid amounts of DNA as compared to two types of control cells, i.e., oligodendrocytes of the same brains and renal tubule cells from the same subjects [37]. It is reasonable that the tetraploid amounts of DNA in the Purkinje cells derive from the endoreduplication process. One of the potential functions for endoreduplication is to ensure production of large amounts of proteins in maintaining cellular homeostasis by increasing the number of gene copies [38,39]. Furthermore, endoreduplication leads to the formation of the lobulated nucleus [40], which exhibits readily in cerebellar Purkinje cells (Figure 8). It is noteworthy that Purkinje cells from the sham surgery control group animals contained PNA. Future research is warranted to explore its origin and its potential relationship with nuclear morphological changes (i.e., nuclear membrane invagination and nuclear lobulation) and endoreduplication as well.

Mechanisms underlying the phenomenon that the polyploid cells are resistant to apoptosis have been described before [32]. First, the anti-apoptotic process in the polyploid cells appears to be applicable in any tissue type; second, a part of proapoptotic gene promoters in the cells in the endocycling process is inhibited; and third, normal endocycling cells have DNA lesions near heterochromatin, indicating that endocycling cells must constantly repress the genotoxic apoptotic response.

A limitation of this study was the small number of animals; specifically, in the formal follow-up study, the sham-operated control group had *n* = 2 and the ischemia group had *n* = 4. Consequentially, when performing statistical analysis on the fluorescence intensity of Mitotracker/CB28 expression, single cells other than those in the animal group were used, namely the ischemic group (*n* = 4) and control group (*n* = 2) of the animals used in the formal follow-up study. In addition, it is arguable that number of the cells exhibiting nuclear TUNEL positivity, nuclear membrane blebbing and a signal of Mitotracker/CB28 in positive control animals (*n* = 1, tissue sample generated 20 years ago [6,7,8]) should have been pooled in the quantitative statistical analysis with the animal in the formal follow-on study (Figure 3). The introduction of tissue sections from the positive control animal helped to link the pilot study with the formal follow-up study in terms of consistency, validity, and reproducibility in the triple immunofluorescence study (TUNEL-Mitotracker/CB28-DAPI) and animal modeling method. Furthermore, in the formal follow-up study, without taking into account the frequency in the positive control samples, there was a significant difference in the frequency of Purkinje cells showing TUNEL positivity between the control and ischemic groups (*p* < 0.001).

Another limitation of this study is that the conclusions regarding the dual characteristics of apoptosis and survival in cerebellar Purkinje cells are based on observations at only one time point (i.e., 4 days after transient global cerebral ischemia) and one animal strain (i.e., adult female Sprague Dawley rats). As described in Section 2, we used tissue blocks collected in previous studies [6,7,8,9,10,11] involving different strains, sexes, and different post-ischemic time points. It is worth noting that there are differences in outcomes following transient global cerebral ischemia between sexes and strains. In adult female Sprague Dawley rats, 20 min of transient global cerebral ischemia resulted in the loss of 50–90% of neurons in the CA1 region of the hippocampus [6], while 40 min of transient global cerebral ischemia resulted in partial or complete damage to the dentate gyrus and partial or complete loss of the hippocampal CA1–CA4 regions, but the animals could survive for 8 days after the ischemia [7,8]. In adult or aged male Fischer 344 rats, 10 min of transient global cerebral ischemia resulted in the loss of up to 90% of neurons in the CA1 region of the hippocampus [9,10,11], while none of the eight total adult male Fischer 344 rats in the first two rounds of the experiment could survive for 8 days after 20 min of transient global cerebral ischemia [unpublished data]. Our approved project only covers the timelines of a few hours or 4 days post-ischemia in adult female Sprague Dawley rats because we only obtained reproducible evidence of apoptotic damage in preliminary experiments and excluded the use of other time points and animal strains. Furthermore, our previous study on apoptotic processes demonstrated caspase 3-mediated apoptosis and estrogen effects in hippocampal CA1 neurons 4 days after transient global cerebral ischemia in female adult Sprague Dawley rats [6]. Another study of ours also showed that aging affected caspase-3-mediated apoptosis on day 3 or later in hippocampal CA1 neurons after transient global cerebral ischemia, but not after 14 days in male Fischer 344 rats [11]. Nevertheless, of interest is whether sex, strain, and time points other than the fourth day after ischemia affect the apoptotic process of cerebellar Purkinje cells.

In conclusion, cerebellar Purkinje cells exhibited dual characteristic of apoptosis and survival four days after transient global ischemia. Key apoptotic indicators included significant mitochondrial loss, expression of active caspase 3, positive TUNEL staining of the nucleus, and nuclear membrane blebbing. Indicators of cell survival included CB28 expression, cytoplasmic compartmentalization and the presence of a certain number of “intact” mitochondria, maintenance of nuclear membrane stability and cell membrane integrity, and transport of NABs/PNAs. These data suggest that certain intracellular events, previously thought canonical to the apoptosis process, may be present in viable Purkinje cells following an ischemic insult. We hypothesize the existence of one or more mechanisms that offer protection against apoptosis, alter the sequence of events leading to apoptotic cell death, or potentially delay apoptosis to a later time point than assessed in this study.

## Data Availability

The original histological and immunofluorescence images are stored under U.S. Food and Drug Administration (FDA) property number 0121206 (associated with the Olympus BX40 microscope) and FDA property number 4005310 (associated with the Zeiss Imager. M2 microscope). The original electron microscopy images are stored under Zeiss Merlin FDA property number 4006034 and FDA property number 0213763. All data are available upon request.
